# Load Magnitude and Locomotion Pattern Alter Locomotor System Function in Healthy Young Adult Women

**DOI:** 10.3389/fbioe.2020.582219

**Published:** 2020-09-16

**Authors:** Kellen T. Krajewski, Dennis E. Dever, Camille C. Johnson, Qi Mi, Richard J. Simpson, Scott M. Graham, Gavin L. Moir, Nizam U. Ahamed, Shawn D. Flanagan, William J. Anderst, Chris Connaboy

**Affiliations:** ^1^Neuromuscular Research Laboratory and Warrior Human Performance Research Center, Department of Sports Medicine and Nutrition, University of Pittsburgh, Pittsburgh, PA, United States; ^2^Biodynamics Laboratory, Department of Orthopedic Surgery, University of Pittsburgh, Pittsburgh, PA, United States; ^3^Department of Nutritional Sciences, University of Arizona, Tucson, AZ, United States; ^4^School of Applied Sciences, Edinburgh Napier University, Edinburgh, United Kingdom; ^5^Exercise Science Department, East Stroudsburg University, East Stroudsburg, PA, United States

**Keywords:** complexity, motor variability, load carriage, motor control, regulation, biomechanics, gait

## Abstract

**Introduction:**

During cyclical steady state ambulation, such as walking, variability in stride intervals can indicate the state of the system. In order to define locomotor system function, observed variability in motor patterns, stride regulation and gait complexity must be assessed in the presence of a perturbation. Common perturbations, especially for military populations, are load carriage and an imposed locomotion pattern known as forced marching (FM). We examined the interactive effects of load magnitude and locomotion pattern on motor variability, stride regulation and gait complexity during bipedal ambulation in recruit-aged females.

**Methods:**

Eleven healthy physically active females (18–30 years) completed 1-min trials of running and FM at three load conditions: no additional weight/bodyweight (BW), an additional 25% of BW (BW + 25%), and an additional 45% of BW (BW + 45%). A goal equivalent manifold (GEM) approach was used to assess motor variability yielding relative variability (RV; ratio of “good” to “bad” variability) and detrended fluctuation analysis (DFA) to determine gait complexity on stride length (SL) and stride time (ST) parameters. DFA was also used on GEM outcomes to calculate stride regulation.

**Results:**

There was a main effect of load (*p* = 0.01) on RV; as load increased, RV decreased. There was a main effect of locomotion (*p* = 0.01), with FM exhibiting greater RV than running. Strides were regulated more tightly and corrected quicker at BW + 45% compared (*p* < 0.05) to BW. Stride regulation was greater for FM compared to running. There was a main effect of load for gait complexity (*p* = 0.002); as load increased gait complexity decreased, likewise FM had less (*p* = 0.02) gait complexity than running.

**Discussion:**

This study is the first to employ a GEM approach and a complexity analysis to gait tasks under load carriage. Reduction in “good” variability as load increases potentially exposes anatomical structures to repetitive site-specific loading. Furthermore, load carriage magnitudes of BW + 45% potentially destabilize the system making individuals less adaptable to additional perturbations. This is further evidenced by the decrease in gait complexity, which all participants demonstrated values similarly observed in neurologically impaired populations during the BW + 45% load condition.

## Introduction

Bipedal ambulation requires the complex integration of multisensory information (optical, somatic and vestibular) that is used to coordinate actions within specific environments in order to achieve goal-directed movement (Alexander, 1992; Warren et al., 2001; Bent et al., 2004a; Pandy and Andriacchi, 2010; Matthis et al., 2017). Perceptions of continuously obtained multisensory information yield opportunities to act (affordances) resulting in a perception-action coupling, with a specific movements success predicated on the modulation (tuning and weighting) of the afferent signals that provide (or fail to) appropriate affordances for the task (Gibson, 1966, 1979; Hollands and Marple-Horvat, 1996; Warren et al., 2001; Bent et al., 2004a,b; Rossignol et al., 2006; Peters et al., 2017). In conjunction with sensory “reafference,” feedforward mechanisms stimulate coordinative structures or muscle synergies that produce a desired movement that achieves a locomotion task goal (Kim et al., 2011; Bizzi and Cheung, 2013; Minassian et al., 2017). Collectively, the reciprocal cooperation of feedback (afferent) and feedforward (efferent) subcomponents executing a locomotion task is known as the locomotor system. The function of the locomotor system reflects the emergent properties of the organization of the degrees of freedom during locomotor tasks, with specific macroscopic pattern of organization being influenced by the confluence of cost functions (i.e., metabolic efficiency and energy dampening), task, organism (including feedback and feedforward processes) and environmental constraints (i.e., gravity, uneven terrain) (Turvey, 1990; Newell and Vaillancourt, 2001; Davids et al., 2003; Sánchez et al., 2016; Caballero et al., 2019; Shafizadeh et al., 2019). Optimal locomotor system function is represented by biomechanical output that is both stable and adaptive to perturbation (Davids et al., 2003; West and Scafetta, 2003; Cusumano and Dingwell, 2013; Seifert et al., 2013). A common perturbation to bipedal locomotion, especially in military populations, is load carriage, especially “combat load” magnitudes of 20–30 kg (Taylor et al., 2016; Krajewski et al., 2020). How the locomotor system accommodates increasing load magnitudes to successfully execute locomotion task goals still remain unclear (LaFiandra et al., 2003; Attwells et al., 2006; Walsh et al., 2018). Thus, measuring the responses of biomechanical variables to the perturbation of additional loading during locomotory tasks provides valuable insight to the global functional state of the locomotor system.

Variability in the observed movement patterns (motor variability) represents the observed variation in a movement solution, when attempting to accomplish the same goal/task, such as using different segment coupling patterns to perform a step (Bernstein, 1967; Latash et al., 2002, 2010; Latash, 2016). The multitude of joints and muscles in the lower extremity lead to a large number of degrees of freedom that lends itself to equifinality; infinite number of movement solutions to accomplish the same task (Bernstein, 1967; Gelfand and Latash, 1998; Latash et al., 2010). A goal equivalent manifold (GEM; equifinality technique) approach seeks to quantify the “good” (plotted tangential to the GEM [δ_T_]) versus “bad” (plotted perpendicular to the GEM [δ_P_]) motor variability to further discriminate optimal performance (known as relative variability [the ratio of “good” motor variability to “bad” motor variability]) (Dingwell et al., 2010; Cusumano and Dingwell, 2013; Sedighi and Nussbaum, 2019). Recent theories have demonstrated that motor variability not only leverages equifinality, making the system more adaptable and stable to perturbation (i.e., overcoming varying terrain or recovering from a slip/trip) (Cusumano and Dingwell, 2013; Dingwell et al., 2017), it also has other cost function benefits (Gates and Dingwell, 2008). Specifically, by capitalizing on a larger workspace (greater relative variability) of movement patterns to perform steady-state (constant locomotion velocity) behaviors, energy can be dispersed through more supportive, anatomical structures, whereas limited motor variability (lower relative variability) may lead to site-specific mechanical overloading (cumulative mechanical stress) that can result in musculoskeletal injury (MSI) (Baida et al., 2018; Nordin and Dufek, 2019). Likewise, motor variability can distribute positive mechanical workloads across a greater number of muscle fibers improving metabolic efficiency by reducing the fatigue of a specific subset of muscle fibers (Gates and Dingwell, 2008).

Regulation of cyclical movements during steady-state behavior such as corrections of stride-to-stride fluctuations further elucidates the state of the locomotor system (Cusumano and Dingwell, 2013). Stride regulation is determined by statistical persistence assessment [alpha coefficients (Dingwell et al., 2017)] of deviations tangential (good variability) [δ_T_] and orthogonal (bad variability) [δ_P_] to the goal manifold (Dingwell et al., 2017). A seminal investigation by Dingwell et al. (2017) demonstrated that elderly individuals classified as low risk fallers and healthy young adults had the same amount of relative variability (ratio of “good” to “bad” motor variability) and used similar stride regulation strategies indicative of a minimal intervention principle (δ_T_α > 1; δ_P_α < 0.5) (Dingwell et al., 2017). Furthermore, it was suggested that changes in stride-regulation strategy to an absolute position control (POS) model [δ_T_α and δ_P_α < 0.5] (Cusumano and Dingwell, 2013) may be the determinant of fall risk (Dingwell et al., 2017). The latter finding was determined with computational modeling (based on a minimum intervention principle) and is still theoretical at this point (Dingwell et al., 2017), but the use of a POS regulation strategy may indicate perception heavily tuned on their exact position, neglecting/overpowering other important information which will impact affordances perception. In the case of military personnel, especially infantry, load carriage is only one perturbation that must be overcome in addition to uneven terrain, enemy threats and decision making. Thus, the quantification of regulation strategy of the system used for stride to stride fluctuations acts as an indirect assessment of the perception-action loop function namely: (i) the ability to (re)calibrate information-action in a dynamic environment, (ii) (re)weighting the relative importance of information sources as they become available, and (iii) modulate based on the relative importance in relation to the successful maintenance of a functionally useful action-response (Cusumano and Dingwell, 2013; Roerdink et al., 2019).

Components of the locomotor system operate/evolve over different time scales and configure in a *heterarchical* organization when functioning optimally (Bak et al., 1987; Turvey, 1990; Bak and Paczuski, 1995; Newell and Vaillancourt, 2001; Davids et al., 2003; Van Orden et al., 2003). A heterarchical organization of a dynamical system is considered to be complex (interaction of many independent subcomponents that yield an emergent behavior) and a perturbation of one subcomponent is less likely to affect the system globally (West and Shlesinger, 1989; Bak and Paczuski, 1995; Marks-Tarlow, 1999; Torre et al., 2007; Torre and Balasubramaniam, 2009). Thus quantification of system complexity indicates the state of dynamical system health (Iyengar et al., 1996; Gisiger, 2001; Goldberger et al., 2005; Hausdorff, 2007; Van Orden et al., 2009; Nourrit-Lucas et al., 2015; Torre et al., 2019) through non-linear signal processing techniques to determine the fractal structure of a time-series, which exhibits self-similarity at different time scales (Stadnitski, 2012). These fractals display long-range correlations, or learning behavior of current iterations from previous iterations (Hausdorff et al., 1995). Time-series structures of gait dynamics (stride length [S_L_] and stride time [S_T_]) that yield long range correlations (pink noise) have been linked to healthy functioning adults (Hausdorff et al., 1997, 1999; Hausdorff, 2007; Delignières and Torre, 2009; Ducharme et al., 2018); however, very strong long-range correlations exhibit over-regularity (brown noise) (Gisiger, 2001). Signals that are completely stochastic (white noise) demonstrate no correlation between strides and have been associated with individuals suffering central neurological impairment (Hausdorff et al., 1997; Hausdorff, 2009; Moon et al., 2016). Moreover, white noise has also been observed when imposing a frequency on cyclical steady-state behavior (Terrier et al., 2005; Terrier and Dériaz, 2012; Hunt et al., 2014; Ducharme et al., 2018; Roerdink et al., 2019). Interestingly, warfighters are encouraged to utilize a walking pattern during a velocity that exceeds the gait transition velocity (GTV), colloquially known as forced marching (FM) that is an unnatural (imposed frequency) gait. Little is known how load magnitude, especially military relevant loads (20–60 kg) (Taylor et al., 2016), and this imposed locomotion affect gait complexity in healthy individuals.

To date a load magnitude perturbation is evidenced only in terms of increased mechanical [greater ground reaction forces (GRF) (Birrell et al., 2007; Seay et al., 2014b) and joint kinetics (Knapik et al., 2004; Seay et al., 2014a,b; Liew et al., 2016; Willy et al., 2016, 2019; Lenton et al., 2019; Loverro et al., 2019; Wills et al., 2019; Krajewski et al., 2020)] and physiological [increased heart rate and ratings of perceived exertion (Simpson et al., 2010, 2011, 2017; Huang and Kuo, 2014)] demands compared to unloaded bipedal ambulation. The majority of these studies consisted of male dominated samples, leaving females underrepresented in load carriage research (Loverro et al., 2019). In addition, females are at twice the risk of MSI (Molloy et al., 2020), with a high incidence (∼78%) of MSI observed during basic combat training (recruits), the majority (30–64%) of those MSI suffered during load carriage conditioning in basic training (Jensen et al., 2019; Lovalekar et al., 2020) suggesting that individuals with little to no experience with load carriage tasks are of greater interest. However, there is a paucity of information regarding the effects of load carriage on motor control (LaFiandra et al., 2003; Attwells et al., 2006; Walsh et al., 2018). Importantly, previous work has focused on average behavior of spatiotemporal gait parameters (LaFiandra et al., 2003; Attwells et al., 2006) and have yet to elucidate key features of a healthy locomotor system such as motor variability, stride to stride regulation and the complexity of the system. Therefore, the purpose of this investigation was to determine the interactive effects of load magnitude and locomotion pattern on motor variability, stride regulation and gait complexity during bipedal ambulation in recruit aged females. It is hypothesized, based on an affordance-based control theory (Davids et al., 2003; Mukherjee and Yentes, 2018) that as load increases and the use of an unnatural (imposed) locomotion (FM) will constrain the locomotor system decreasing the number of affordances available which will be reflected by the reduction in relative variability (the ratio of “good” motor variability to “bad” motor variability). Likewise, increases in load and utilization of FM will lead to stricter regulation strategies. Lastly, individuals gait complexity will decrease as load increases and during the execution of the FM locomotion pattern.

## Materials and Methods

### Ethics Statement

All participants were read and signed informed consent that has been approved by the Institutional Review Board (IRB) of The University of Pittsburgh. They were notified of potential risks and benefits associated with participation in the study.

### Subjects and Protocol

Eleven healthy, recreationally active young adult females (see Table 1 for participant characteristics) participated in this study. Recreationally active was defined as engaging in moderate physical activity a minimum of two times a week for at least 30 min, similar to comparable recruits. Moreover, women novice to load carriage and forced marching were chosen to represent a female recruit population, replicating initial exposure to load carriage tasks. Subjects were screened to exclude individuals who reported spine and lower extremity musculoskeletal injury, neurological disorder or pregnant.

**TABLE 1:** Subject Characteristics and exercise status.

**ID**	**Age (yr)**	**Wt (kg)**	**Ht (m)**	**BF%**	**Ses/Wk**	**Min/Ses**	**Min/Wk**	**Modes of exercise**	**LC Exp**	**CC**
S1	27	56.5	1.57	21.2	4	45	180	running, boxing, cycling	N	S*
S2	27	62.4	1.69	32.8	3	90	270	running, rowing	N	O^†^
S3	21	62.1	1.57	31.7	3–5	30	90–150	running, walking	N	I
S4	21	50.8	1.53	23.1	6	90	540	cardio, weightlifting	Rec	O
S5	24	47.6	1.55	7.8	6	60–90	360–540	running, cycling, swimming	Rec	O^†^
S6	28	72.6	1.65	40.4	2–3	45	90–135	elliptical, yoga, hiking, kayaking	N	S
S7	25	70.6	1.68	34.4	3–5	30–40	90–200	running, calisthenics	N	O^†^
S8	24	60.9	1.64	33.8	3–5	60	180–300	running, cycling, pilates, zumba, weightlifting	N	O^†^
S9	24	52.9	1.64	14.5	5–6	60–90	300–540	running, weightlifting	Mil	S
S10	25	54.4	1.63	30.3	5	60	300	running, weightlifting, soccer	N	S*
S11	24	81.0	1.72	21.8	6	40	240	running, swimming	Rec	O^†^
Mn	24.5	61.1	1.6	26.5	4.5	58.6		-	-	-

Wt = Weight; Ht = Height. Ses = Sessions; Wk = Week; Min = Minutes. BF% = Body fat percentage. LC Exp = Load Carriage Experience; Rec = Recreational; Mil = Military; N = None. CC = Complexity Classification (at baseline); S = Suboptimal; O = Optimal; I = Impaired. Mn = Mean; * = Observed improvements in complexity classification from baseline (FMBW, RN + 25% and FM + 25% conditions only); † = Maintained optimal complexity classification (FMBW and RN + 25% conditions only).

The procedures for this investigation have been previously described in detail (Krajewski et al., 2020). Briefly, participants ran (RN) and forced marched (FM) on a instrumented split-belt treadmill (Bertec Corporation, Columbus, OH, United States) for 1 min at three different loaded conditions: Bodyweight (BW), plus an additional 25% of BW (BW + 25%), and plus an additional 45% of BW (BW + 45%) [which represents 20–30 kg “combat” loads in average young adult females (Taylor et al., 2016)] at 10% above their GTV (BW: 2.08 ± 0.25 m/s; BW + 25%: 2.02 ± 0.22 m/s; BW + 45%: 1.93 ± 0.23 m/s). All participants wore provided combat boots (Speed 3.0 Boot, 5.11 Tactical, Irvine, CA, United States) to control for influences of footwear on kinematics (Telfer et al., 2017) and loaded conditions were executed with an anterior-posterior loaded weight vest (Short Plus Style Vest, MIR, United States) to control for effects of center of mass (COM) location (Seay et al., 2014a; Loverro et al., 2019). All trials were randomized by load condition and then by locomotion pattern. Participants were given up to 5 min between each trial to control for effects of fatigue. During RN trials, participants were instructed to move “naturally” or how they felt most comfortable to maintain treadmill velocity. For FM trials, participants were instructed to maintain a walking gait regardless of the treadmill velocity. Each trial yielded ∼130 strides (120–180) dependent on the locomotion pattern and velocity. Prior to familiarization and data collection, participants filled out an activity questionnaire and body composition was assessed with dual energy X-ray absorptiometry (DXA) [Lunar iDXA, General Electric, Boston, MA, United States].

### Data Collection and Processing

**FIGURE 1:**
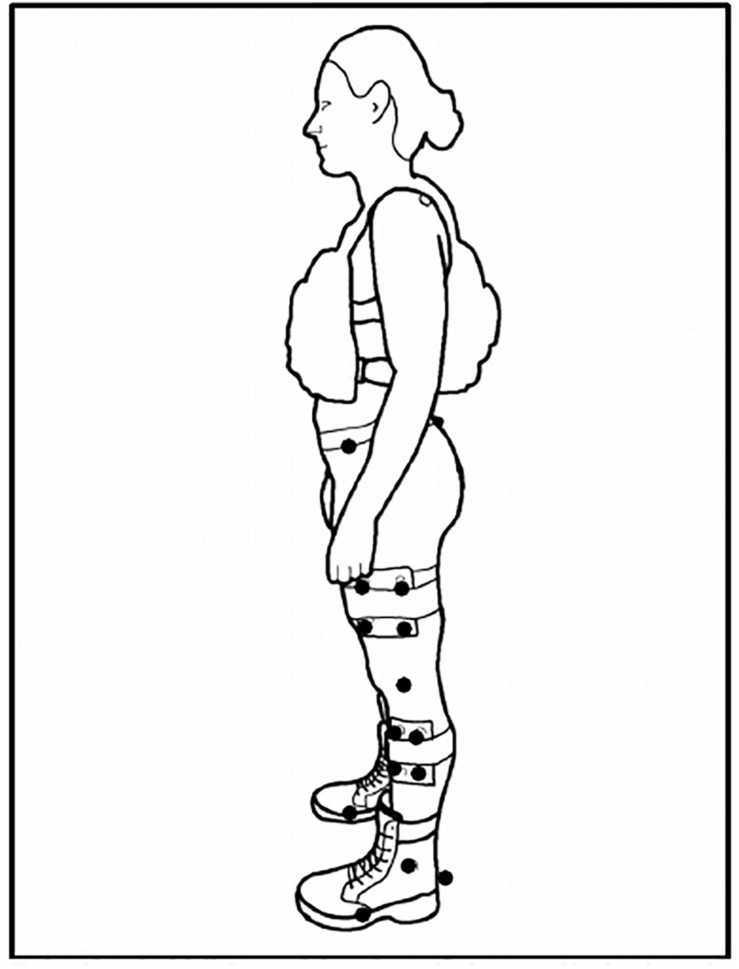
Subject Set-Up. Exemplar set up of a participant with their +45% load in the anterior-posterior weight vest. Solid dots represent retroreflective markers. Markers at the medial/lateral epicondyles (knee) and medial/lateral malleoli (ankle) removed after static calibration trial capture. Markers at the calcaneus and 1st/5th metatarsophalangeal (MTP) joints defined the foot segment.

Three retro-reflective markers were placed on each boot (calcaneus, 1st and 5th metatarsophalangeal [MTP] joints) [see Figure 1 for subject experimental set up]. Kinematic data was collected via 12 infrared cameras (Vicon Motion Systems Ltd., Oxford, United Kingdom) sampling at 100 Hz. Kinetic data was collected via an instrumented split-belt treadmill sampling at 1000 Hz that was synchronized with the motion analysis system. Using the Vicon Nexus^®^ 2.0 software (Vicon Motion Systems Ltd., Oxford, United Kingdom), a custom labeling template was created for the marker configuration used in the study. Once all static and motion trials were reconstructed, the labeling template was used to auto label the static trials captured for each load condition (BW, BW + 25% and BW + 45%) which were then used to auto label their respective motion trials (RN and FM). Gap filling methods in Nexus 2.0 were used to correct any breaks in trajectory data due to marker occlusion. Data was then exported, and post processed in Visual 3D (C-Motion Inc., Germantown, MD, United States). Further analysis [GEM decomposition and Detrended Fluctuation Analysis (Atlas Collaboration et al., 2014)] was conducted with custom Matlab^TM^ 2019a (Mathworks, Inc., Natick, MA, United States) scripts. Kinematic and kinetic data were filtered with a second order Butterworth low-pass filter (cut-off frequencies of 6 Hz and 40 Hz for the kinematic and kinetic signals, respectively). Heel strike was defined as the time when vertical component of the ground reaction force exceeded a 50N threshold.

The following variables were calculated: Stride length (S_L_) was computed as the distance covered from heel strike to ipsilateral heel strike; Stride time (S_T_) was computed as the time elapsed from heel strike to ipsilateral heel strike; Stride speed (S_S_) was computed as the quotient of S_L_/S_T_; Velocity (v) was computed as the average S_S_ over all *n* strides of a time-series. Average values (Means), standard deviations (SD) and DFA scaling exponents (α-value) were calculated for S_L_, S_T_ and S_*S*_ across all trials.

### Goal Equivalent Manifold Decomposition

Methods utilized for GEM decomposition have been described in detail by Dingwell et al. (2010). However, to further elaborate the process: firstly, S_L_ and S_T_ time-series for each trial was normalized to unit variance [dividing by its own standard deviation]. A specific operating point was computed for S_T_ as Eq. (1):

(1) $$S_{T}^{*}={<S_{T}>}_{n}$$

Where <■> represents the average across all *n* strides of the time series. The specific operating point for S_L_ was computed as Eq. (2):

(2) $$S_{L}^{*}=v\,S_{T}^{*}$$

Here *v* represents the velocity of the treadmill for that specific trial. The new centered operating point was then computed as Eqs. (3) and (4):

(3) $$S_{T\,n}^{\prime }=S_{T_{n}}-S_{T}^{*}$$

and

(4) $$S_{L_{n}}^{\prime }=S_{L_{n}}-S_{L}^{*}$$

Lastly, deviations tangential to the goal manifold were represented as δ_T_ and deviations perpendicular to the goal manifold were represented as δ_P_. These deviations were calculated with a linear coordinate transformation as Eq. (5):

(5) [δTδP]=11+v2⁢[1v-v1]⁢[STn′SLn′]

Where, the σ of δ_T_ and δ_P_ were determined for each load and locomotion condition. Relative variability was calculated as the ratio between σδ_T_/σδ_P_. Therefore, a relative variability magnitude of 1 represents equal amounts of “good” versus “bad” variability; <1 represents more “bad” variability; and >1 represents more “good” variability. Additionally, DFA scaling exponents (α) were computed for δ_T_ and δ_P_. Scaling exponents for δ_T_ and δ_P_ are interpreted as follows: α < 0.5 represents anti-persistence (alteration in one direction more likely followed by an alteration in opposite direction); α > 0.5 represents statistical persistence (alteration in one direction more likely followed by an alteration in same direction); and α = 0.5 represents uncorrelated (alteration in one direction has same likelihood of being followed by alteration in either direction) (Dingwell et al., 2010, 2017).

### Complexity Analysis

Complexity analysis was executed utilizing fractal methods, specifically DFA (Peng et al., 1993; Hausdorff et al., 1995; Delignières et al., 2006; Stadnitski, 2012) on S_L_ and S_T_ gait variables (∼130 consecutive strides). Refer to the aforementioned references for greater detail but briefly: DFA creates a one-dimensional signal *x*(*i*), *i* = 1,…, L, where *x* is the initial signal of size L, and an integrated signal (*x*) is calculated according to the Eq. (6):

(6) $$Y\,\left(k\right)=\sum_{i=1}^{k}\left(B\,\left(i\right)-B_{a\,v\,g}\right)$$

where *B*_avg_ is the mean value of the signal (*B* = signal value at specific time point). The unified time series *Y* is then divided into segments (boxes that don’t overlap) of length *l*, and the linear approximation *Y*_*l*_ is then obtained through a least-squares fit of each segment separately (trend of each section).

The mean fluctuation (root mean square) of the incorporated and detrended time-series is computed using Eq. (7):

(7) $$F\,\left(l\right)=\sqrt{\frac{1}{L}\,\sum_{k=1}^{L}{\left(Y\,\left(k\right)-Y_{l}\,\left(k\right)\right)}^{2}}$$

The aforementioned calculations are repeated for a range of *l*. The goal of this analysis is to identify the relation between *F(l)* and the size of segment *l* because this relationship serves as an indicator of a scaling phenomenon. In general, *F*(*l*) increases with increases in the range of segment *l*. A double plot logarithmic graph (*log* (*F*(*l*) vs *log*_*l*_) is then formed, and this graph is used to acquire the scaling exponent (α). A linear dependency implies the existence of self-fluctuations, and *F*(*l*) which is the slope of line outlines the scaling α exponent, which increases with *l* based on a power law, as detailed in Eq. (6):

(8) $$F\left(l\right)-l^{\alpha }=>log(\left(F\left(l\right)\right)-\alpha \times log\left(l\right)$$

DFA ultimately yields a scaling exponent (α) which represent the correlational structure of the signal. White noise (uncorrelated or completely stochastic) is represented as α = 0.5; Pink noise (positive long-range correlations) is represented as α = 1.0; Brown noise (persistent long-range correlations or too much regularity) is represented as α = 1.5 (Peng et al., 1995). Classifications based upon a range of α were employed to provide greater clarity as values are rarely the exact values listed above. “Suboptimal self-organization” was represented by α < 0.75; “Optimal self-organization” was represented by α = 0.75 – 1.30; “Impaired self-organization” was represented by α > 1.30. These values were based upon previously established ranges that classified populations (healthy, elderly, and impaired) as either white, pink or brown noise (Hausdorff et al., 1996, 1997, 1999; Ravi et al., 2020). The values attained during the RN with BW condition was considered the baseline because it is the natural locomotion pattern used 10% above GTV and unperturbed by an external load (no added load carriage). Change classifications were then determined for each individual (change from the baseline condition) as either “positive change” in complexity (“Suboptimal self-organization” or “Impaired self-organization” to “Optimal self-organization”), “negative change” (“Optimal self-organization” to “Suboptimal self-organization” or “Impaired self-organization”), “no change positive” (“Optimal self-organization” to “Optimal self-organization”) and “no change negative” (“Suboptimal self-organization” or “Impaired self-organization” to “Suboptimal self-organization” or “Impaired self-organization”).

### Statistical Analysis

Descriptive statistics (mean and SD) were reported for all the variables. In order to determine interactive effects of load and locomotion on relative variability and gait complexity a two-way repeated measure analysis of variance (RMANOVA) for Load × Locomotion (3 × 2) was conducted separately. Additionally, to further elucidate findings regarding relative variability, tangential and perpendicular variability were assessed within locomotion pattern with a 3 × 2 (Load × Direction) RMANOVA. If interactions were significant, simple main effects were performed (paired *t*-tests for locomotion/direction stratified by load and RMANOVA for load stratified by locomotion/direction). If no significant interaction was observed, only main effects were analyzed. *Post hoc* analysis using Bonferroni-corrected pairwise comparisons were conducted when necessary.

To determine interactive effects of load and locomotion on stride regulation a three-way Load × Locomotion × Direction (3 × 2 × 2) RMANOVA was conducted on DFA scaling exponents of δ_T_ and δ_P_. If a significant three-way interaction was observed then two-way RMANOVAs were conducted for load by locomotion (3 × 2), load by direction (3 × 2) and locomotion by direction (2 × 2). If a two-way interaction was observed, then simple main effects were analyzed (RMANOVA for load and paired *t*-tests for locomotion and direction). If no significant two-way interaction was observed, only main effects were analyzed. *Post hoc* analysis using Bonferroni-corrected pairwise comparisons were conducted when necessary. Lastly, if no significant three-way interaction was observed, only main effects were analyzed. *Post hoc* analysis using Bonferroni-corrected pairwise comparisons were conducted when necessary.

Partial eta squared (η^2^*_P_*) was calculated as a measure of effect size given the within-subject design (Bakeman, 2005; Richardson, 2011), with magnitudes of effect interpreted as: 0.01–0.085 (small effect); 0.09–0.24 (moderate effect); and > 0.25 (large effect) (Cohen et al., 2003). Additionally, frequencies of complexity classifications and change classifications are reported to qualitatively examine individual responses. The alpha level was set at 0.05 (*p* ≤ 0.05).

## Results

### Relative Variability

See Table 2 for mean and SD of all GEM related outcomes. There was no significant interaction between load and locomotion for relative variability of motor control (*F*_2_,_20_ = 0.167, *p* = 0.85, *η*^2^*_P_* = 0.02). Load had a significant influence on relative variability reducing the number of successful or “good” movement solutions, exemplified as relative variability magnitude decreasing as load magnitude increased confirmed by the main effect of load (*F*_2_,_20_ = 5.50, *p* = 0.01, *η*^2^*_P_* = 0.36); with *post hoc* analysis revealing BW + 45% (1.28 ± 0.05) being significantly (*p* = 0.02) less than BW (1.55 ± 0.07). Additionally, FM demonstrated a more relative variability compared to running indicated by the main effect of locomotion (*F*_1_,_10_ = 8.90, *p* = 0.01, *η*^2^*_P_* = 0.47) with estimated marginal means for FM (1.53 ± 0.08) being greater than RN (1.27 ± 0.04).

**TABLE 2:** GEM outcomes (mean ± standard deviation).

	**Run**			**Forced marching**		
**Variable**	**BW**	**+25%**	**+45%**	**BW**	**+25%**	**+45%**
S_L_	1.52 ± 0.20	1.48 ± 0.17	1.38 ± 0.15	1.70 ± 0.14	1.63 ± 0.13	1.54 ± 0.12
S_T_	0.74 ± 0.04	0.75 ± 0.03	0.74 ± 0.04	0.84 ± 0.05	0.82 ± 0.05	0.83 ± 0.07
RV	1.41 ± 0.33	1.27 ± 0.18	1.14 ± 0.17	1.69 ± 0.40	1.47 ± 0.37	1.42 ± 0.23
δ_T_ (V)	1.13 ± 0.09	1.10 ± 0.06	1.05 ± 0.07	1.20 ± 0.08	1.15 ± 0.09	1.15 ± 0.06
δ_P_ (V)	0.83 ± 0.13	0.88 ± 0.08	0.94 ± 0.08	0.74 ± 0.13	0.81 ± 0.13	0.82 ± 0.09
δ_T_ (α)	0.91 ± 0.29	0.55 ± 0.41	0.35 ± 0.51	0.57 ± 0.38	0.43 ± 0.40	0.09 ± 0.38
δ_P_ (α)	0.68 ± 0.22	0.39 ± 0.30	−0.01 ± 0.51	0.21 ± 0.43	0.11 ± 0.40	−0.21 ± 0.36

*S_*L*_ = Stride Length (meters); S_*T*_ = Stride Time (seconds). RV = Relative Variability (σδ_*T*_/σδ_*P*_). δ_*T*_ (V) = Tangential variability; δ_*P*_ (V) = Perpendicular variability. δ_*T*_ (α) = Tangential coordinate scaling exponent; δ_*P*_ (α) = Perpendicular coordinate scaling exponent. BW = Body Weight (no additional load).*

The interaction between load and direction during RN was not statistically significant (*F*_2_,_20_ = 2.33, *p* = 0.12, *η*^2^*_*p*_* = 0.19). There was no significant main effect of load (*F*_2_,_20_ = 3.05, *p* = 0.07, *η*^2^*_*p*_* = 0.23). While not significant, as load increased mean tangential (“good”) variability decreased (BW = 1.13 ± 0.10, BW + 25% = 1.10 ± 0.06, BW + 45% = 1.06 ± 0.07) and mean perpendicular (“bad”) variability increased (BW = 0.84 ± 0.13, BW + 25% = 0.88 ± 0.08, BW + 45% = 0.94 ± 0.08). However, regardless of load, tangential variability was always greater evidenced by the main effect of direction (*F*_1_,_10_ = 60.91, *p* < 0.001, *η*^2^*_*p*_* = 0.86), with estimated marginal means revealing variability along the tangential (1.10 ± 0.01) was greater than along the perpendicular (0.88 ± 0.02).

**FIGURE 2:**
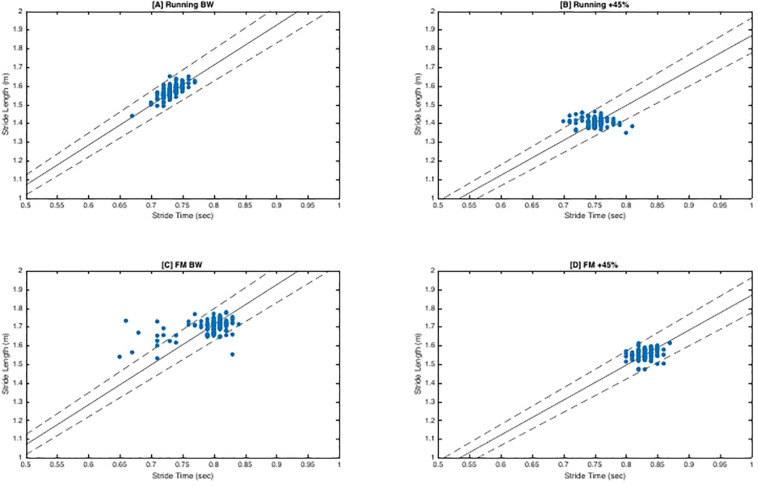
Goal Equivalent Manifold (GEM) Exemplar Plots. Exemplar plots (S4) represent a time series of consecutive strides (∼156 strides). Solid dots represent the different combinations of SL and ST for each stride. The solid line represents the goal manifold, which in this case is the velocity of the treadmill. Therefore, the assumed goal of the participant is to maintain horizontal velocity so they do not drift off the end of the treadmill belt. The dashed lines superior and inferior to the solid line represent the ±5% error of the goal manifold. Dots that are tangential (along the solid goal manifold) are variations that still achieve the task goal (‘good’ variability). Dots that are perpendicular to the solid goal manifold line are variations that fail to achieve the goal manifold. Perpendicular coordinates will result in the participant moving forward or backward on the treadmill belt. (A,B) demonstrate more tangential variability as indicated by the larger spread along the goal manifold. Conversely, (C,D) exhibit a much tighter formation indicative of less variation and stricter stride regulation. Furthermore, in contrast of (A), (C) exhibits more stride variants that lie beyond the ±5% error range. Not surprisingly, this participant had complexity classifications of ‘optimal’ and ‘suboptimal’ for (A,C) respectively.

There was no significant interaction between load and direction for FM (*F*_2_,_20_ = 1.79, *p* = 0.19, *η*^2^*_*p*_* = 0.15). There was no main effect of load (*F*_2_,_20_ = 2.75, *p* = 0.09, *η*^2^*_*p*_* = 0.22). While not significant, as load increased mean tangential (“good”) variability decreased slightly (BW = 1.20 ± 0.08, BW + 25% = 1.15 ± 0.08, BW + 45% = 1.15 ± 0.06) and mean perpendicular (“bad”) variability increased (BW = 0.74 ± 0.013, BW + 25% = 0.81 ± 0.12, BW + 45% = 0.81 ± 08). Lastly, regardless of load, tangential variability was always greater than perpendicular variability indicated by the main effect of direction (*F*_1_,_10_ = 90.10, *p* < 0.001, *η*^2^*_*p*_* = 0.90), with estimated marginal means revealing tangential variability (1.17 ± 0.02) was greater than perpendicular variability (0.79 ± 0.02). See Figure 2 for exemplar plots of S_L_ and S_T_ combinations along the goal manifold.

### Scaling Exponents (α): *S_T_ & S_p_*

See Table 2 for all means and SD of scaling exponents. There was no significant 3-way interaction between load, locomotion and direction (*F*_2_,_20_ = 1.96, *p* = 0.17, *η*^2^*_p_* = 0.16). As load increased control of stride-to-stride fluctuations became more strict (and therefore corrected more quickly) evidenced by the main effect of load (*F*_2_,_20_ = 8.87, *p* = 0.002, *η*^2^*_p_* = 0.47), with *post hoc* analysis revealing that BW (0.6 ± 0.08) was significantly (*p* = 0.02) greater than BW + 45% (0.04 ± 0.11). Additionally, running exhibited less control of stride-to-stride fluctuations indicated by the main effect of locomotion (*F*_1_,_10_ = 8.57, *p* = 0.02, *η*^2^*_p_* = 0.46), with estimated marginal means revealing that running (0.46 ± 0.07) was greater than FM (0.19 ± 0.08). Lastly, “bad” variations (perpendicular to the goal manifold) were controlled and corrected more quickly than “good” variations (tangential to goal manifold) as evidenced by the main effect of direction (*F*_1_,_10_ = 67.12, *p* < 0.001, *η*^2^*_p_* = 0.87), with estimated marginal means revealing that persistence along the tangential (0.47 ± 0.06) was greater than the perpendicular (0.18 ± 0.07).

### Complexity Analysis (α): S_L_, S_T_

See Table 3 for mean and SD of scaling exponents. There was no significant interaction between load and locomotion for S_L_ (*F*_2_,_20_ = 0.03, *p* = 0.97, *η*^2^*_*p*_* = 0.003). As load magnitude increased, gait complexity decreased independent of locomotion pattern as evidence by the main effect of load (*F*_2_,_20_ = 8.74, *p* = 0.002, *η*^2^*_*p*_* = 0.4 = 7), with *post hoc* pairwise comparisons revealing BW (0.69 ± 0.06) was significantly (*p* = 0.02) greater than BW + 45% (0.1 ± 0.16). Additionally, FM reduced gait complexity compared to running independent of load magnitude indicated by the main effect of locomotion (*F*_1_,_10_ = 7.59, *p* = 0.02, *η*^2^*_*p*_* = 0.43), with estimated marginal means revealed running (0.59 ± 0.06) was greater than FM (0.23 ± 0.13).

**TABLE 3:** Complexity outcomes (mean ± standard deviation) and class frequency (# of occurrences).

		**Run**			**Forced marching**		
**Variable**		**BW**	**+25%**	**+45%**	**BW**	**+25%**	**+45%**
S_L_ (α)		0.88 ± 0.31	0.63 ± 0.26	0.27 ± 0.63	0.49 ± 0.42	0.27 ± 0.46	−0.07 ± 0.55
S_T_ (α)		1.04 ± 0.50	1.09 ± 1.02	0.15 ± 0.54	0.29 ± 0.62	0.04 ± 0.53	−0.34 ± 0.59
‘O’	S_L_	6	4	2	4	3	0
	S_T_	6	1	2	1	1	0
‘S’	S_L_	4	7	9	7	8	11
	S_T_	2	6	9	9	10	11
‘I’	S_L_	1	0	0	0	0	0
	S_T_	3	4	0	1	0	0

*S_*L*_ = Stride Length; S_*T*_ = Stride Time. α = alpha coefficient derived from detrended fluctuation analysis. White Noise = 0.5 (‘Suboptimal self-organization’ [‘S’] represented as < 0.75). Pink Noise = 1 (‘Optimal self-organization’ [‘O’] represented as 0.75 – 1.30). Brown Noise = 1.5 (‘Impaired self-organization’ [‘I’] represented as >1.30).*

There was no significant interaction between load and locomotion for S_T_ (*F*_2_,_20_ = 0.43, *p* = 0.36, eta = 0.10). The increase in load magnitude decreased gait complexity independent of locomotion pattern as evidenced by the main effect of load (*F*_2_,_20_ = 6.52, *p* = 0.007, *η*^2^*_*p*_* = 0.40), with *post hoc* analysis revealing that BW (0.67 ± 0.10) was greater (*p* = 0.03) than BW + 45% (−0.10 ± 0.19). Lastly, FM reduced gait complexity compared to running independent of load magnitude as indicated by the main effect of locomotion (*F*_1_,_10_ = 9.66, *p* < 0.001, *η*^2^*_*p*_* = 0.75), with estimated marginal means revealing running (0.76 ± 0.11) was greater than FM (−0.003 ± 0.15).

**FIGURE 3:**
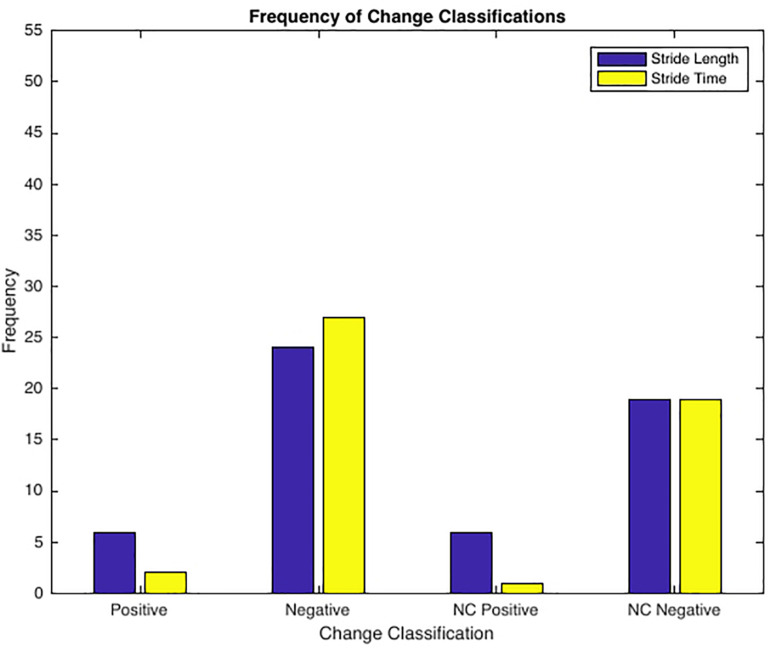
Frequency of Change Classifications. Positive Change = ‘Suboptimal’ to ‘Optimal’ or ‘Impaired’ to ‘Optimal’ (only 2 subjects, 50% of incidences 1 subject); Negative Change = ‘Optimal’ to ‘Suboptimal’ or ‘Optimal’ to ‘Impaired’; No Change Positive = ‘Optimal’ to ‘Optimal’ (2 subjects accounted for 59% of all incidences); No Change Negative = ‘Suboptimal’ to ‘Suboptimal’, ‘Suboptimal’ to ‘Impaired’, ‘Impaired’ to ‘Impaired’, or ‘Impaired’ to ‘Suboptimal’.

See Table 3 for frequency of observed complexity classifications by condition and Figure 3 for the frequency of each classification change of all conditions combined. For S_L_ and S_T_ 43.64% and 49.09% of the observed changes from baseline were negative changes (“Optimal self-organization” to “Suboptimal’ or ‘Impaired.” Only two participants demonstrated a positive change with 63% of those occurrences accounted for by one participant. Positive change classifications only occurred at the BW + 25% load condition and not the BW + 45%. Two of the participants with positive changes from baseline reported having prior experience in a multitude of exercise modalities that would improve lower limb muscular endurance and anaerobic conditioning (see Table 1). For S_L_ only 10.91% of changes were classified as “no change positive” with 58% of the occurrences accounted for by two individuals. Both individuals performed a greater variety of exercise modalities including resistance training and trained more frequently and for longer periods of time. Lastly, only a single subject demonstrated an “Impaired” classification at baseline (RN at BW). This participant trained for the shortest durations (30 min maximum) only 2–3 times a week (see Table 1 for more detailed characteristics).

## Discussion

The objectives of this investigation were to determine the interactive effects of load magnitude and locomotion pattern on motor variability, stride-to-stride control/regulation and complexity. Load magnitude significantly altered relative variability independent of locomotion pattern evidenced by the significant main effect of load. As load increased relative variability decreased with BW + 45% (1.28σ) having 21% less relative variability compared to BW (1.55σ), suggesting individuals are better able to leverage either the system degeneracy, or indeed, redundancy of coordinative patterns employed to execute a stride during unloaded bipedal ambulatory tasks. The higher relative variability ratios are achieved by a greater variance tangential the goal manifold (“good” variability) and a reduction in variance perpendicular to the goal manifold (“bad” variability) [see Figure 2]. Importantly, these findings alone do not necessarily indicate more stable performance (task execution), but that during unloaded conditions there is a greater workspace of solutions that can be utilized to accomplish the goal task (maintain velocity). Coupled with the observed complexity scaling exponents ∼1 for S_L_/S_T_ at BW and BW + 25% load conditions suggest that individuals display an optimally organized locomotor system fully leveraging equifinality. Furthermore, the lack of significant differences between BW and BW + 25% load conditions indicates that the latter load condition is insufficient to impose a constraint on the locomotor system that results in altered motor variability. At BW + 45%, the locomotor system has a diminished ability to leverage “good” motor variability. The lack of observed “good” variability, may limit energy dispersion across multiple structures up the kinetic chain when energy is highest [due to increased forces from load (Birrell et al., 2007; Lloyd, 2010; Liew et al., 2016; Willy et al., 2016, 2019; Krajewski et al., 2020)] increasing cumulative mechanic stress MSI risk (Nordin and Dufek, 2019; Baggaley et al., 2020).

Contrary to the proposed hypothesis, the FM (RV = 1.52) resulted in 19.6% greater relative variability than running (RV = 1.27). However, this finding alone should be interpreted with caution; the greater variability simply denotes a larger set of motor solutions observed for FM compared to running. Similar observations were made by Black et al. (2007) demonstrating greater motor variability in adolescents with down syndrome compared to aged-matched neurotypical adolescents, indicating that during unperturbed steady-state gait, those with down-syndrome needed to utilize their full movement solution potential in order to maintain velocity (Black et al., 2007). In addition, Black et al. (2007) demonstrated that the greater motor variability exhibited during unperturbed gait suggested a locomotor system operating closer to the action boundaries that would be more likely to fail (fall) with additional pertubrations (Black et al., 2007). Thus, greater relative variability alone may not indicate more task performance stability or motor-system health. In females during FM there is a greater contribution of frontal plane moments (adduction/abduction) to knee total joint moment, indicative of potentially deleterious movements (Krajewski et al., 2020). Although a greater range of movement solutions were utilized during FM compared to running, they may have included more movement solutions that while successful in executing the goal task (maintaining velocity), are maladaptive/deleterious with respect to joint/tissue health (Zabala et al., 2013; Asay et al., 2018; Baggaley et al., 2020; Krajewski et al., 2020). Future work should focus on elucidating the movement pattern structures (segment couplings) in more detail, which coincide with relative variability, its magnitude and structure.

Additionally, the unfamiliarity of the task for this sample (they had little to no experience with FM) may have demonstrated a greater exploration of state-space as a consequence of learning to perform this locomotion pattern (Newell and Vaillancourt, 2001; Pacheco and Newell, 2017). The greater relative variability exhibited by FM compared to running may be indicative of a locomotor system trying to adapt to improve mechanical efficiency or reduce pain sensations associated with each stride. By obstructing the natural bifurcation of locomotion (participants executing FM at a velocity 10% above their GTV), the system is in search of an order parameter (segment coupling pattern) that adheres to cost function (decrease metabolic cost/increase mechanical efficiency) of the task control parameter (horizontal velocity) (McGinnis and Newell, 1982; Newell and Vaillancourt, 2001; Davids et al., 2003; Caballero et al., 2019; Shafizadeh et al., 2019). Consequently, the observed complexity for FM was α = 0.11 (“suboptimal”), supporting the notion that the observed greater relative variability in FM compared to running, represented a state-space exploratory behavior. In conjunction with the low system complexity, the locomotor system is less stable to additional perturbations. Whilst imposed locomotion (FM) and load carriage are themselves perturbations dynamic military environments present even more perturbations (uneven terrain, enemy threats, orders from local commanders). If motor learning occupies a large percentage of the system workspace, it potentially results in the failure to identify external perturbations [due to competition over feedback/feedforward resources (Woollacott and Shumway-Cook, 2002; Al-Yahya et al., 2011; De Sanctis et al., 2014)] making the locomotor system less stable and more susceptible to failure (slips/trips/falls, identification of threats). Future research should compare novice and experienced individuals with load carriage to determine if there is a difference in relative variability and complexity while completing ambulatory tasks with load.

Stride-to-stride regulation demonstrated less control for unloaded conditions as evidenced by the main effect of load (*p* = 0.002). The BW + 45% load condition exhibited (δ_T_α = 0.22, δ_p_α = −0.11) significantly more regulation than BW (δ_T_α = 0.74, δ_p_α = 0.45). Alpha coefficient (α) values less than.5 indicate statistical anti-persistence, representing much stricter control because the subsequent stride variation is more likely to be the opposite composition (combination of S_L_ and S_T_) than the previous. Thus, movements were corrected much quicker and more often with the addition of substantial (BW + 45%) load carriage independent of locomotion pattern. Moreover, the main effect of direction (*p* < 0.001) exhibited more strict regulation perpendicular to the manifold (“bad” variability) compared to tangential (“good” variability) [see Table 1 for δ_T_ (α) and δ_p_ (α) means]. The combination of less control of “good” variability and more control of “bad” variability [resembling an ideal minimum intervention principle (MIP) model [δ_T_α > 1; δ_p_α < 0.5] (Cusumano and Dingwell, 2013)] is indicative of a stride-regulation strategy of a healthy system recognizing movement variations that impede the execution of the task goal (maintain velocity). Further, the “system controller” minimally intervenes, minimizing control effort theoretically freeing up system capacity for other components of the locomotor system (Cusumano and Dingwell, 2013). In fact, the BW results (δ_T_α = 0.74, δ_p_α = 0.45) of this investigation were similar to stride regulation findings of healthy adults (δ_T_α = ∼0.90, δ_p_α = ∼0.42) by Dingwell et al. (2017). If the individual were unhealthy, a failure to properly regulate “bad” motor variability potentially results in them unable to successfully execute the task. However, as load increased both δ_T_α and δ_p_α decreased indicating a change in stride-regulation strategy that reflected an absolute position control (POS) model [δ_T_α and δ_p_α < 0.5] (Cusumano and Dingwell, 2013), postulating that maximal control effort was used thus reducing the capacity of other locomotor system components (Cusumano and Dingwell, 2013).

Locomotion pattern also affected stride-to-stride regulation independent of load, with FM (δ_T_α = 0.36, δ_p_α = 0.04) demonstrating stricter control compared to running (δ_T_α = 0.60, δ_p_α = 0.35). Even “good” motor variability (δ_T_α) was tightly regulated for FM evidenced by the δ_T_α < 0.5. Once again, the stride-regulation strategy utilized for FM at all load conditions more closely mimicked that of a POS model (Cusumano and Dingwell, 2013). A stricter regulation of stride-to-stride intervals coupled with greater relative variability of FM at BW + 45% compared to RN BW + 45% may further evidence the participant learning by exploring state-space and freezing/unlocking different degrees of freedom quickly (Bernstein, 1967; Sporns and Edelman, 1993; Newell and Vaillancourt, 2001). Thus in response to the load and imposed locomotion perturbations a structural reorganization of the locomotor system occurs, with potentially greater reliance on supraspinal input that disrupt feed forward mechanisms of gait (further supported by the observed α < 0.5 for FM of S_L_ and S_T_) due to the competition over feedback/feedforward resources (Woollacott and Shumway-Cook, 2002; Al-Yahya et al., 2011; De Sanctis et al., 2014). It is likely that the perturbations associated with FM and load elicit more system capacity dedicated to control in a relatively unperturbed state (walking on a treadmill) potentially overpowering other system components important to navigating dynamic environments. This may have important consequences to military populations, as loaded ambulatory tasks are often undertaken in dynamic circumstances requiring the integration of multiple information sources to understand the context within which the action takes place, i.e., quickly navigating across an open area of the battle space while aiming and firing a weapon.

When assessing complexity of gait dynamics (S_L_ and S_T_) there was a main effect of load (*p* = 0.002 and *p* = 0.007), with complexity decreasing as load increased. The BW + 45% condition (S_L_α = 0.1; S_T_α = −0.1) exhibited significant less complexity than BW (S_L_α = 0.69; S_T_α = 0.67). Whilst, group mean comparison demonstrated a decrease in complexity, the group mean for BW still represents a “suboptimal” organization (α < 0.75) (Hausdorff et al., 1997; Hausdorff, 2007). However, when observing the individual results, eight of the participants had “optimal” complexity or.75 < α < 1.30 during BW load conditions (see Tables 1, 3). Moreover, only five participants had “optimal” complexity for BW + 25% and every participant exhibited “suboptimal” complexity for the BW + 45% load condition. “Optimal” complexity or scaling exponents (α) ∼1 represent long-range correlations (pink noise) that is indicative of skilled performance and utilization of prior stride information (i.e., proprioceptive) to influence future strides (Hausdorff et al., 1996, 1997; Delignières and Torre, 2009; Nourrit-Lucas et al., 2015). Likewise, the fractal structure of “optimal” represents an independence of fluctuations at different time scales meaning a perturbation of one system component will not likely affect the global system (locomotor system as a whole) (West and Shlesinger, 1989; Bak and Paczuski, 1995; Marks-Tarlow, 1999; Torre et al., 2007). Therefore, the data observed within the present study indicates that a load carriage magnitude of at least BW + 45% reduces the system stability and adaptability, predisposing the system to failure (falling) in the presence of additional perturbations (i.e., increased fall risk with uneven terrain) in females with limited/no load carriage experience. Importantly, BW + 45% for this female sample was 26.6 ± 4.7kg, which represents a typical combat load (20 kg) (Taylor et al., 2016), a load used during operations that will bombard individuals with perturbations (terrain obstacles[debris], enemy threats, officer commands/questions) that if not actioned correctly could result in serious MSI or death. The latter suggests the need for further research to determine if experience/training with load carriage improves system complexity that can better handle additional adaptations.

In addition to a main effect of load, locomotion patterns also affected the complexity of S_L_ and S_T_ (*p* = 0.02 and *p* < 0.001, respectively). As hypothesized, the natural locomotion pattern running (S_L_α = 0.59; S_T_α = 0.76), exhibited greater complexity than FM (S_L_α = 0.23; S_T_α < −0.01). Considering that running is the extant locomotion observed at a velocity exceeding GTV, it is not surprising more participants (seven) had “optimal” complexity during RN conditions (BW and BW + 25% only). Five of the participants did have “optimal” complexity for FM but at the BW and BW + 25% conditions only. These five participants (S1, S7, S8, S10, and S11) engaged in moderate amounts of exercise time per week and engaged in a multitude of exercise modalities that included a form of anaerobic conditioning and/or strength training (see Table 1). Furthermore, one participant (S3) had an “impaired” complexity at baseline (running at BW). Interestingly, this participant performed the least amount of exercise time per week and engaged in the least varied modes of exercise [see Table 1]. Thus, the movement poverty in terms of time and coordination diversity, may impact system adaptability as reflected in the “impaired” and “suboptimal” complexity during minimally perturbed steady-state behaviors. Moreover, the complexity outcomes demonstrated varying individual responses highlighting its potential utility as a mechanical “biomarker” of the current state of the locomotor system and training adaptation response. Future research should compare complexity of different fitness level groups (i.e., highly aerobic versus highly anaerobic fit individuals) during loaded ambulatory tasks to further elucidate characteristics of “optimal” performers.

The primary limitations of this study are its small, sex specific (female) sample and the number of consecutive data points (∼130). While this investigation cannot make inferences about sex specific responses to load carriage in the absence of a male cohort, females are an under represented population in load carriage literature (Loverro et al., 2019). Therefore, findings regarding motor variability, stride regulation and gait complexity of this study can only be generalized to healthy recruit-aged females (18–33 years old) whom are novices to load carriage and forced marching. Future research should compare males versus females and recruit versus experienced individuals (deployable soldiers) to determine if the unfamiliarity to the tasks and equipment were confounding the observed responses to load magnitude and locomotion pattern. Likewise, it is advised to perform fractal analysis with a minimum of 512 consecutive data points (Delignières et al., 2006), however, 128 consecutive strides has been determined to be within 6% of the actual scaling value (Hausdorff et al., 1997). In addition, performing steady-state behavior with load carriage while minimizing the effects of fatigue is difficult beyond several minute trials. Moreover, our findings on the effect of locomotion pattern on gait complexity are similar to investigations regarding imposed frequencies and complexity (Delignieres et al., 2004; Ducharme et al., 2018). Nevertheless, while not ideal for complexity, the ∼130 consecutive data points is a robust time-series for GEM decomposition (Dingwell et al., 2010) and provides the first quantitative data on load magnitude’s influence on gait complexity. Lastly, upon the completion of the study we learned that one participant (S5) completed the experimental protocols with a distal avulsion of the semitendinosus. Interestingly, this participant exhibited “optimal” complexity for running at BW and BW + 25%. Although alone this data is inconclusive and may represent an isolated incident, but it does suggest that gait complexity of S_L_ and S_T_ may be an inappropriate factor to assess musculoskeletal health. The complexity of S_L_ and S_T_ specifically may only represent the global function of the locomotor system at achieving the task goal.

In conclusion, there are no interactive effects of load magnitude and locomotion pattern on motor variability, stride regulation and gait complexity. But load and locomotion do independently alter the function of the locomotor system. As load increases there is a reduction in relative variability (good:bad motor variability), gait complexity (α < 0.5) and strides become more tightly controlled. FM further reduces gait complexity and mimics stride regulation strategies of BW + 45% load conditions. Moreover, despite more relative variability for FM compared to running, this appears to be a consequence of state-space exploration, as supported by the increased stride control/error correction and “suboptimal” complexity. While complexity of S_L_ and S_T_ may be indicative of locomotor system function in terms of achieving a task goal (maintaining horizontal velocity in the case of this investigation), the variability/complexity of these factors do not appear to represent the health of the musculoskeletal system (i.e., state of joint/tissue health and whether an injurious movement pattern is being used). Therefore, additional order parameters (different gait variables) should be investigated to identify a marker of global MSI risk. Likewise, research should be conducted with longer trials to confirm the complexity findings of this investigation and elucidate the role of fatigue. Soldiers ultimately operate in dynamic environments with lots of perturbations under substantial load carriage where poor movement/slips, trips and fall can produce MSI or even death. The findings of this study indicate that locomotor system function is altered by FM and BW + 45% load, resulting in reduced motor variability and a system with less stability/adaptability that is potentially more susceptible to failure with additional perturbations.

## Data Availability Statement

The raw data supporting the conclusions of this article will be made available by the authors, upon reasonable request.

## Ethics Statement

The studies involving human participants were reviewed and approved by the Institutional Review Board of University of Pittsburgh. The patients/participants provided their written informed consent to participate in this study.

## Author Contributions

KK, QM, NA, and CC analyzed the data. CC, WA, and KK designed the study and wrote the manuscript. KK, CJ, and DD were responsible for the data collection and data processing. RS, SG, GM, QM, SF, and WA contributed to writing and review and editing the manuscript. All authors contributed to the article and approved the submitted version.

## Conflict of Interest

The authors declare that the research was conducted in the absence of any commercial or financial relationships that could be construed as a potential conflict of interest.
